# Device neo-endothelialization after left atrial appendage closure: the role of cardiac computed tomography angiography

**DOI:** 10.1007/s10554-021-02206-2

**Published:** 2021-03-17

**Authors:** Roberto Galea, Christoph Gräni

**Affiliations:** Department of Cardiology, Inselspital, Bern University Hospital, University of Bern, CH-3010 Bern, Switzerland

## Editorial

Percutaneous left atrial appendage closure (LAAC) has emerged as a valid therapeutic option for prevention of thromboembolic events in patients with non-valvular atrial fibrillation and contraindication for oral anticoagulation. Both, long-term efficacy and safety have been proved in two randomized clinical trials and in many multicenter registries. However, similar to other cardiovascular metallic implants, LAAC devices have the potential to develop device related thrombus (DRT) on the non-endothelialised free surface [1], with the consequence of possible thromboembolic events [2].

The neo-endothelialization (NE) of LAAC devices is incompletely understood. Small animal studies showed at 45 days after LAAC an organized neo-endocardial layer covering the atrial surface of the Watchman device [3]. After 90 days, an additional fibrous pannus tissue was observed covering the neo-endothelium [3]. Therefore, it is recommended in the real-world setting to install an antithrombotic therapy for a similar period in order to prevent formation of DRT in patients with newly implanted LAAC devices. However, some observations suggest that in humans complete NE of the device atrial surface may take longer [1], and rising concerns regarding the adequate duration of antiplatelet therapy after procedure. Therefore, a reliable imaging method to assess the sealing of LAAC device and to confirm the complete NE is needed.

In this context, we would like to congratulate on the pilot imaging study presented by Lindner et al. [4] where a new definition of complete NE of LAAC devices, as evaluated by cardiac computed tomography angiography (CCTA), has been proposed. The authors reported the 6-month CCTA follow-up finding from a monocentric observational prospective cohort, including 53 consecutive patients submitted to clinically indicated LAAC. Incomplete NE was defined as presence of residual left atrial appendage (LAA) patency in the absence of peridevice leak (PDL). As a consequence, the 17 of 53 (32%) who showed PDL were excluded from this analysis. Out of the remaining 36 patients, 20 patients showed a residual patent LAA, reflecting an incomplete NE rate of 56% at 6 months after implantation. This study shows several novelties. Firstly, it is the first study to propose a scientifically rigorous method to assess device NE by CCTA. In 2018, Garnier et al. reported a similar definition of NE by using a different imaging modality (i.e. transesophageal echocardiography [TEE]) instead of CCTA to exclude PDL [5]. Subsequently, Sivasambu et al. proposed to exclude PDL by using CCTA; however the assessment of LAA patency was performed semi-quantitatively comparing the density of residual LAA with density of myocardium (no HU values were proposed as cut-offs) [6]. Secondly, Lindner et al. introduced the concept that residual LAA patency may be the effect of three different mechanisms such as: (a) incomplete NE, (b) PDL, or (c) both. So far, several studies have only reported the first two mechanisms as a potential residual patency. The authors therefore decided to exclude PDL from the analysis since the contributing role of incomplete NE in LAA patency could not be assessed in the presence of concomitant PDL. Thirdly, Lindner et al. observed a rate of complete NE of 44%, and much lower than expected based on animal studies. However, comparing the results of the NE rate observed by the authors with those reported by larger studies [7, 8] have to be interpreted with caution, given the heterogenous definitions and different CCTA timing (Table 1). Yet, it is worth noting that all studies have reported a significant percentage of incomplete NE surrogate (7–30%). This is particularly important considering that studies comparing different imaging modality (i.e. TEE and CCTA) to assess NE are missing.

**Table 1:** Selected studies assessing neo-endothelialization using CCTA after LAA closure

Study	Number of patients	CCTA timing (months)	Definition of incomplete NE	Incomplete NE (Excluding patients with PDL) (%)	Incomplete NE (Including all patients) (%)
Sivasambu et al. [6]	46	1.5	Residual PA* in the absence of PDL	35	22
Qamar et al. [8]	102	3	Residual PA† with visible diffusion of contrast through the atrial surface of device	12	7
Lindner et al. [4]	53	6	Residual PA¶ in the absence of PDL	56	30

*CCTA* cardiac computed tomography angiography, *NE* neo-endothelialization, *PDL* peri-device leak, *PA*,patent appendage, *LAA* left atrial appendage, *HU* hounsfield unit, *LA* left atrial

^*^Adjudicated semi-quantitatively, comparing density of LAA with myocardium density (no cut-off were mentioned)

^†^Defined as LAA density < 100 HU or < 25% LA density

^¶^Defined as LA density – LAA density ≤ 50 HU

Although this study has the important merit to propose a new method to assess NE of LAAC devices, enthusiasm to routinely apply this approach should be tempered by several caveats. First, the authors defined LAA as patent if the attenuation of the left atrium (LA) exceeded that of the LAA by 50 Hounsfield Units (HU) or less, and therefore using a different definition from previous studies (i.e. LAA attenuation either ≥ 100UH or ≥ 25% of that measured in LA) [7, 8]. Of note, the latter cut-offs were validated in large populations undergoing both TEE and CCTA and showed a 100% sensitivity and a 66.7% specificity of detecting residual patent LAA in CCTA compared to the TEE [8]. CCTA assessment by means of the above cut-offs, therefore, seems to better identify residual LAA patency compared to TEE, although its clinical significance is still unclear. Furthermore, a difference of only 50 UH between LA and LAA attenuation (sufficient according to Lindner et al. to define LAA as sealed) may inherit the risk to reduce CCTA sensitivity in detecting patent LAA, especially in well contrasted LA where high attenuation may be observed in both LA and residual LAA despite a significant absolute difference. Moreover, a clear definition of PDL as evaluated by CCTA still needs to be standardized, as it is unclear if the gap detected at the device margins has to be extended for the entire length of the device or even for a portion of that continuing then inside it. Yet, the method proposed by the authors does not allow to assess NE in presence of concomitant PDL. To overcome this issue, either the definition of “fabric leak” reported by Qamar et al. (visible diffusion of contrast through the non-endothelialised atrial surface of device) [8] or the enhancement defect observed on the atrial surface of devices may represent valid NE surrogates.

Nevertheless, the study of Lindner et al. is an important step forward to the development of an accurate NE assessment method and reinforces the role of CCTA as the primary imaging method to follow-up patients after LAAC. A standard definition of NE with a broad consensus in the scientific community would be needed in the future. Therefore, different definitions of NE need to be tested in larger multicenter studies with serial CCTAs in order to better clarify the duration of device healing process, and to assess whether incomplete NE correlates to adverse long-term clinical events.
